# A Genetically Truncated RGD-Containing Peptide rLj-RGD4 Exhibits Potent In Vivo Antitumor Activity via Induction of Multi-Pathway Apoptosis and EGFR-Targeted Signaling Suppression

**DOI:** 10.3390/molecules31081266

**Published:** 2026-04-11

**Authors:** Yuyao Song, Huijie Yan, Yuebin Zhang, Jingyu Zhang, Li Lv, Jihong Wang

**Affiliations:** 1 School of Life Sciences, Liaoning Normal University, Dalian 116081, China; yolanda_songyy@hotmail.com (Y.S.); huijie.y@hotmail.com (H.Y.); 2 Department of Pharmacology, College of Pharmacy, Dalian Medical University, Dalian 116044, China; yuebinz@hotmail.com (Y.Z.); jingyuzhang2000@hotmail.com (J.Z.)

**Keywords:** rLj-RGD4 peptide, antitumor activity, apoptosis, internalization, EGFR

## Abstract

Although the parental recombinant protein rLj-RGD3 exhibits antitumor activity, it carries immunogenicity risks owing to its large molecular size (13.5 kDa). We generated a genetically truncated mutant, rLj-RGD4 (6.27 kDa, four RGD motifs), which inhibited B16 melanoma cell proliferation, migration, and invasion in vitro. However, the in vivo efficacy and mechanisms of action remain unclear. Here, B16 xenograft mice were treated with rLj-RGD4 (5, 10, and 20 μg/kg i.p. daily for 14 days). Tumor growth was measured, and histopathology/apoptosis was evaluated using hematoxylin and eosin (HE), Masson’s dye, Hoechst, and TUNEL staining. Apoptotic pathways (mitochondrial, death receptor, and MAPK) were analyzed via Western blotting, whereas endocytosis mechanisms were explored using inhibitors (filipin III, NaN_3_, cytochalasin D), and EGFR (epidermal growth factor receptor) interactions via fluorescence co-localization and phosphoprotein assays. The results demonstrated dose-dependent tumor growth inhibition (21.60–89.26% volume reduction, 41.03–86.51% weight reduction), with histological evidence of tissue loosening, fibrosis, and apoptosis. rLj-RGD4 induced apoptosis by activating the mitochondrial (Bax/Bcl-2 upregulation), death receptor (caspase-8 activation), and MAPK (JNK/p38 phosphorylation) pathways. Internalization was blocked by NaN_3_ and cytochalasin D, indicating actin-dependent macropinocytosis. Direct EGFR binding was confirmed, accompanied by reduced EGFR expression and the inhibition of FAK/AKT/Src signaling. In conclusion, rLj-RGD4 exerts potent in vivo antitumor activity via two mechanisms: induction of multi-pathway apoptosis and EGFR-targeted suppression of pro-survival signaling. RGD4 exerts its antitumor function in vivo by targeting and co-internalizing with EGFR.

## 1. Introduction

Melanoma, which originates from cutaneous, mucosal, and uveal melanocytes, is the most aggressive form of skin cancer and is characterized by early metastasis, high mortality, and resistance to conventional chemotherapy [1,2,3]. Despite advances in the understanding of its molecular pathogenesis, the etiology of melanoma remains incompletely defined, and effective curative treatments are limited. Recent studies have highlighted the key oncogenic drivers (e.g., *BRAF* and *NRAS* mutations) and signaling networks (e.g., MAPK and PI3K/AKT pathways) involved in melanoma progression, fostering the development of targeted therapies, such as kinase inhibitors, immunotherapies, and molecularly targeted agents [4,5,6,7].

A critical hallmark of melanoma malignancy is the ability to invade the extracellular matrix (ECM) and metastasize, which is tightly regulated by cell-ECM adhesion [8]. Integrins, a family of heterodimeric cell adhesion receptors, have been implicated in tumor cell migration, invasion, and angiogenesis by mediating interactions with ECM components (e.g., fibronectin and vitronectin) [9,10]. The Arg-Gly-Asp (RGD) motif, a conserved tripeptide sequence, serves as a key ligand for integrins (e.g., αvβ3, αvβ5), making RGD-containing peptides promising candidates for disrupting tumor cell adhesion and signaling [11,12,13].

The recombinant peptide rLj-RGD4, derived from the parental protein rLj-RGD3, is a novel RGD-based agent with four tandem RGD motifs and reduced molecular weight (6.27 kDa), potentially minimizing the immunogenicity risks associated with larger proteins [14]. Our previous in vitro studies demonstrated that rLj-RGD4 inhibited B16 melanoma cell proliferation, migration, and invasion while disrupting actin cytoskeleton organization. However, the in vivo antitumor efficacy and underlying mechanisms, particularly the interactions with other oncogenic receptors, remain uncharacterized.

The epidermal growth factor receptor (EGFR/HER1/ErbB1), a 170 kDa transmembrane tyrosine kinase receptor, is frequently overexpressed in melanoma and drives tumor growth by activating downstream pathways (e.g., RAS/RAF/MEK/ERK and PI3K/AKT) [15,16,17]. EGFR signaling is tightly regulated by endocytic trafficking, and dysfunctional internalization has been linked to sustained mitogenic signaling and therapeutic resistance [18]. Notably, cross-talk between RGD-based integrin inhibitors and EGFR has been reported, with certain RGD peptides demonstrated to modulate EGFR localization and activity [19,20]. This raises the hypothesis that rLj-RGD4 exerts dual-targeting effects on integrins and EGFR, thereby enhancing its antitumor potency.

We systematically evaluated the in vivo antitumor activity of rLj-RGD4 in B16 melanoma xenograft models and investigated its molecular mechanisms, with a focus on confirming tumor growth inhibition and histological changes, elucidating the apoptotic signaling pathways activated by rLj-RGD4, characterizing the cellular internalization routes, and exploring the potential interactions with EGFR. These findings provide preclinical evidence for the development of rLj-RGD4 as a dual-target anticancer agent with an improved therapeutic index.

## 2. Results

### 2.1. Purification and Identification of rLj-RGD4 Peptide

**Figure 1:**
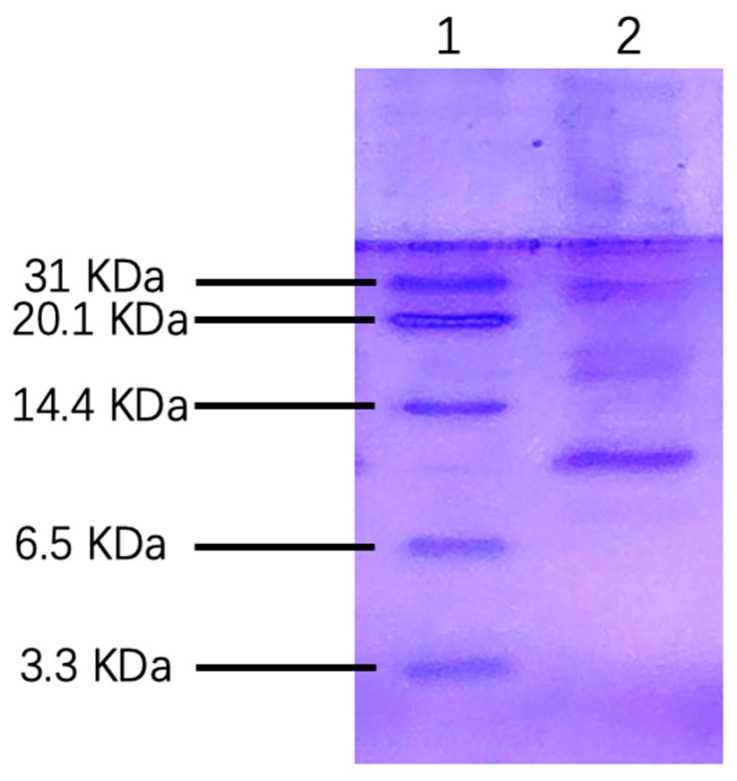
Tricine-SDS PAGE of purifying of rLj-RGD4 peptide (Line 1: Marker; Line 2: rLj-RGD4 peptide).

Tricine SDS–PAGE was used to characterize the rLj-RGD4 peptide purified using nickel-nitrilotriacetic acid (Ni-NTA) affinity chromatography. As shown in Figure 1 the electrophoretic profile revealed a single distinct band corresponding to a molecular weight of 6.27 kDa, which was consistent with the theoretical calculations. The purity of the soluble rLj-RGD4 peptide was estimated to exceed 95%, based on densitometric analysis. Notably, the electrophoretic mobility of the peptide exhibited a slight lag relative to the expected position, which was attributed to its basic isoelectric point. This is a common phenomenon in SDS–PAGE for proteins with extremely charged properties. These results confirmed the successful purification of the target peptide with high homogeneity, providing a reliable sample for subsequent functional studies.

### 2.2. Tumor Suppressor Effect of rLj-RGD4 Peptide in B16 Melanoma-Homozygous Mice

#### 2.2.1. Dose-Dependent Antitumor Efficacy and Safety Profile of rLj-RGD4 Peptide

**Figure 2:**
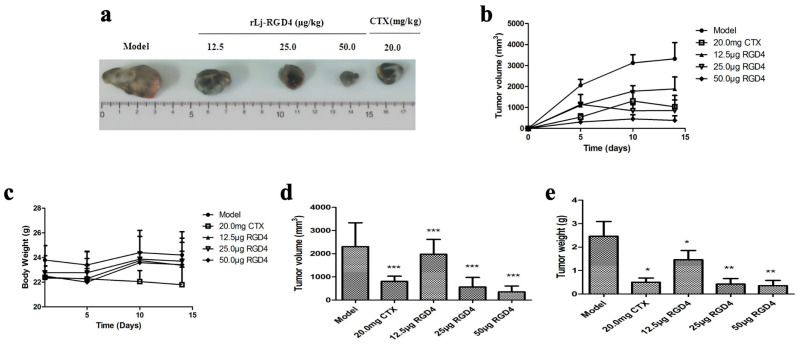
(**a**) Effect of rLj-RGD4 peptide on the size of tumor volume in B16 melanoma mice. (**b**) Effect of rLj-RGD4 peptide on the growth trend of tumor volume in B16 melanoma mice (*n* = 8). (**c**) Effect of rLj-RGD4 peptide on the body weight of B16 melanoma-bearing mice (*n* = 8). (**d**) Effect of rLj-RGD4 peptide on the tumor volume of B16 melanoma-bearing mice (*n* = 8, compared with control, *** *p* < 0.001). (**e**) Effect of rLj-RGD4 peptide on the tumor weight of B16 melanoma-bearing mice (*n* = 8, compared with control, * *p* < 0.05, ** *p* < 0.01).

Tumor-bearing mice in the model and rLj-RGD4 peptide groups (12.5, 25.0, and 50.0 μg/kg) exhibited slight body weight gain with normal activities at the end of the experiment, whereas mice in the CTX group showed significant weight loss (Figure 2c), reduced food intake, and decreased activity, indicating substantial toxic effects of CTX. Tumor growth kinetics (Figure 2a,b) revealed a rapid and continuous volume increase in the model group, significantly exceeding that in the other groups. In contrast, rLj-RGD4 treatment dose-dependently attenuated tumor growth, with the high-dose group (50.0 μg/kg) even demonstrating negative tumor growth in late-stage experiments. Medium and high doses of rLj-RGD4 showed tumor inhibition efficacy comparable to CTX (20.0 mg/kg), with the high-dose group outperforming the CTX group in terms of antitumor activity. The rLj-RGD4 peptide demonstrated significant dose-dependent inhibition of B16 melanoma growth in tumor-bearing mice following 2 weeks of treatment (Table 1, Figure 2d). The rLj-RGD4 peptide exhibited significant dose-dependent inhibition of tumor weight in B16 melanoma-bearing mice (Table 2, Figure 2e).

#### 2.2.2. Effect of rLj-RGD4 Peptide on Histopathological Changes in Tumor Tissues of B16 Melanoma-Bearing Mice

**Figure 3:**
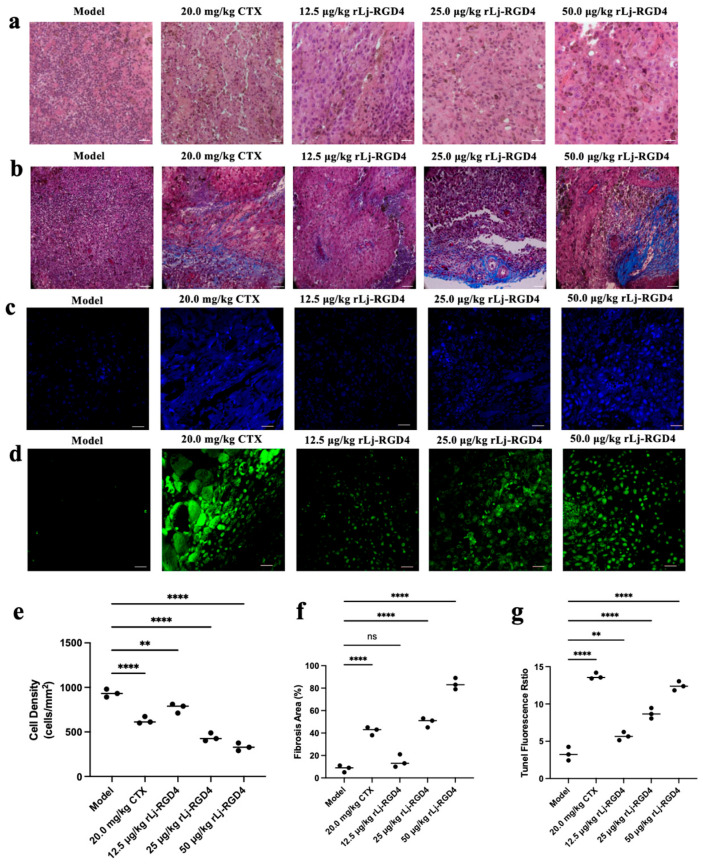
(**a**) H-E staining of tumor tissues after treatment with rLj-RGD4 peptides and CTX (OLYMPUS digital imaging microscope; Tokyo, Japan, 400×; scale: 10 µm). (**b**) Masson trichrome staining of tumor tissues after treatment with rLj-RGD4 peptides and CTX (OLYMPUS digital imaging microscope 400×; scale: 10 µm). (**c**) Hoechst 33258 staining of tumor tissues after treatment with rLj-RGD4 peptides and CTX (Zeiss laser scanning confocal microscope; Oberkochen, Baden-Wurttemberg, Germany, 400×; scale: 10 µm). (**d**) TUNEL staining of tumor tissues after treatment with rLj-RGD4 peptides and CTX (Zeiss laser scanning confocal microscope, 400×; scale: 10 µm). (**e**) Tumor cell density (HE staining) was significantly reduced in a dose-dependent manner after rLj-RGD4 administration. (**f**) Fibrosis area (Masson’s trichrome staining) was markedly increased in rLj-RGD4-treated groups in a dose-dependent manner. (**g**) TUNEL-positive apoptotic cell ratio was significantly elevated in rLj-RGD4-treated groups, with a dose-dependent effect. Data are mean ± SD. ** *p* < 0.01, **** *p* < 0.0001 vs. Model group; ns, not significant.

HE staining of the B16 melanoma tissues revealed pink cytoplasm and blue-purple nuclei (Figure 3a). The model group exhibited uniformly stained, densely packed tumor cells with compact tissue architecture and regular nuclear morphology. In contrast, all treatment groups demonstrated significant histological alterations, including reduced cellular density, irregular cell morphology, disorganized tissue architecture, and heterogeneous nuclear features with chromatin condensation. These pathological changes displayed dose-dependent progression in the rLj-RGD4-treated groups, with the highest dose group demonstrating comparable or superior effects to CTX treatment. These findings confirmed the potent, dose-dependent antitumor activity of the rLj-RGD4 peptide at microgram-level doses against B16 melanoma.

Masson’s trichrome staining revealed distinct collagen deposition patterns in the B16 melanoma tissues (Figure 3b). Although control tumors showed dense cellularity with minimal blue-stained collagen fibers (indicating the absence of fibrosis), rLj-RGD4-treated groups and the CTX-positive control exhibited dose-dependent fibrotic changes: reduced nuclear density and significantly increased blue-stained collagen areas. The high-dose rLj-RGD4 group demonstrated comparable fibrotic induction to the CTX treatment group, suggesting that rLj-RGD4 effectively promotes tumor fibrosis, a potential mechanism contributing to its antitumor effects through structural disruption of the tumor microenvironment.

Hoechst 33258 staining revealed distinct apoptotic features in the B16 melanoma tissues (Figure 3c). Compared with the control group, the rLj-RGD4-treated groups and the CTX-positive control exhibited dose-dependent increases in blue fluorescence intensity, corresponding to enhanced chromatin condensation and DNA fragmentation, which are characteristic hallmarks of apoptosis. The high-dose rLj-RGD4 group demonstrated pro-apoptotic effects comparable to those of the CTX treatment, suggesting that rLj-RGD4 effectively induced tumor cell apoptosis through a dose–response mechanism.

The TUNEL assay demonstrated a significant induction in apoptosis in B16 melanoma tissues (Figure 3d). Compared with the control group, rLj-RGD4-treated groups and CTX-positive control demonstrated dose-dependent increases in green fluorescence intensity, reflecting enhanced 3′-OH DNA fragment labeling by TdT-mediated dUTP incorporation. The high-dose rLj-RGD4 group achieved apoptotic effects comparable to those of CTX treatment, confirming rLj-RGD4’s ability to trigger tumor cell apoptosis through DNA fragmentation in a concentration-dependent manner.

#### 2.2.3. Signaling Pathways Involved in rLj-RGD4 Peptide-Induced Apoptosis in Tumor Cells of B16 Melanoma-Bearing Mice

**Figure 4:**
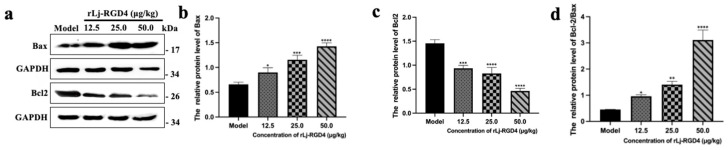
(**a**) The protein levels of Bax and Bcl-2 in tumor tissues. (**b**) The relative gray value of Bax in tumor tissues of each group, and GAPDH was used as a control. * *p* < 0.05; *** *p* < 0.001; **** *p* < 0.0001. (**c**) The relative gray value of Bcl-2 in tumor tissues of each group; GAPDH was used as a control. *** *p* < 0.001; **** *p* < 0.0001. (**d**) The relative gray value of Bcl-2/Bax in tumor tissues of each group; GAPDH was used as a control. * *p* < 0.05, ** *p* < 0.01, **** *p* < 0.0001.

Western blot analysis demonstrated that rLj-RGD4 regulated the expression of key apoptotic regulators in B16 melanoma tissues in a dose-dependent manner (Figure 4). Compared with the control group, the rLj-RGD4-treated group exhibited a gradual upregulation of pro-apoptotic Bax and downregulation of anti-apoptotic Bcl-2, resulting in a significant dose-dependent increase in the Bax/Bcl-2 ratio. This change in the protein expression profile suggests that rLj-RGD4 inhibits tumor growth by inducing apoptosis via the mitochondria-regulated apoptotic pathway.

**Figure 5:**
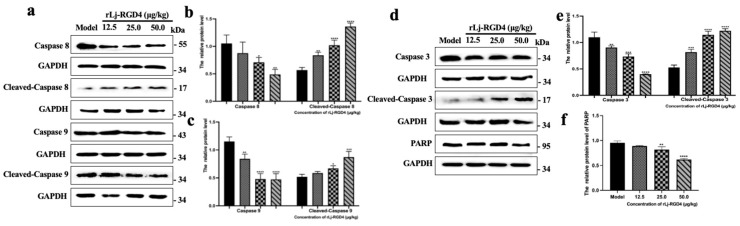
(**a**) The protein levels of caspase 8/9 and cleaved-caspase 8/9 in tumor tissues. (**b**) The relative gray value of caspase 8 and cleaved-caspase 8 in tumor tissues of each group. GAPDH was used as a control; * *p* < 0.05, ** *p* < 0.01, **** *p* < 0.0001. (**c**) The relative gray value of caspase 9 and cleaved-caspase 9 in tumor tissues of each group, GAPDH was used as a control. * *p* < 0.05, ** *p* < 0.01, *** *p* < 0.001, **** *p* < 0.0001. (**d**) The protein levels of caspase 3, PARP, and cleaved-caspase 3 in tumor tissues. (**e**) The relative gray value of caspase 3 and cleaved-caspase 3 in tumor tissues of each group, and GAPDH was used as a control. ** *p* < 0.01, *** *p* < 0.001, **** *p* < 0.0001. (**f**) The relative gray value of PARP in tumor tissues of each group, GAPDH was used as a control. ** *p* < 0.01, **** *p* < 0.0001.

Western blot analysis demonstrated that the rLj-RGD4 peptide activated the exogenous apoptotic pathway in B16 melanoma tissues via a caspase-8-mediated mechanism in a dose-dependent manner (Figure 5). The expression of caspase-8/9/3 was significantly decreased in all treatment groups, and its cleaved form correspondingly increased, resulting in subsequent downregulation of the expression of the substrate PARP. Activated caspase-8 initiates two distinct apoptotic cascades: initiation of the mitochondrial pathway via Bax-mediated cytochrome c release and subsequent caspase-9 activation, and the direct cleavage of effector caspase-3. The rLj-RGD4 peptide induces apoptosis in tumor cells via multiple pathways, including the mitochondrial-mediated apoptotic pathway and the death receptor signaling pathway, ultimately inhibiting tumor growth in B16 melanoma-bearing mice.

**Figure 6:**
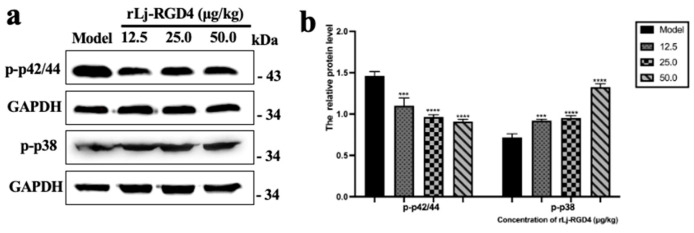
(**a**) The protein levels of p-p42/44 and p-p38 in tumor tissues. (**b**) The relative gray value of p-p42/44 and p-p38 in tumor tissues of each group, GAPDH was used as a control. *** *p* < 0.001, **** *p* < 0.0001.

In addition, Western blot analysis revealed that rLj-RGD4 peptide treatment significantly modulated MAPK signaling pathways in B16 melanoma tissues (Figure 6). The peptide downregulated the expression of phosphorylated p42/44 MAPK (Erk1/2) in a dose-dependent manner, which regulates cell proliferation and differentiation, and concurrently upregulated phosphorylated p38 MAPK, a stress-activated kinase involved in the induction of apoptosis. These findings suggest that the rLj-RGD4 peptide exerts its antitumor effects through dual regulation of MAPK pathways: suppression of proliferative signaling via p42/44 MAPK inhibition and promotion of apoptotic signaling through p38 MAPK activation.

### 2.3. Mechanisms of rLj-RGD4 Peptide Action on B16 Cells and Its Effect on EGFR

#### 2.3.1. Inhibitory Effect of rLj-RGD4 Peptide on B16 Cell Proliferation

**Figure 7:**
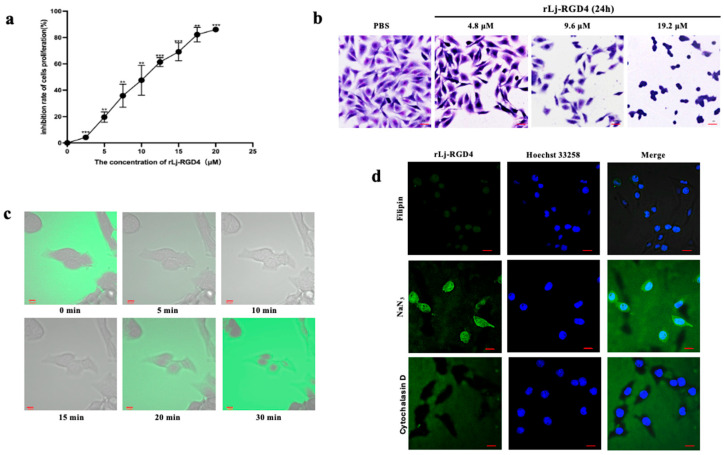
(**a**) The inhibitory effect of rLj-RGD4 on the proliferation of B16 cells using the CCK-8 method. ** *p* < 0.01, *** *p* < 0.001. (**b**) Examination of the effect of rLj-RGD4 on the proliferation of B16 cells by Giemsa staining (OLYMPUS digital imaging microscope; Tokyo, Japan, 400×; scale: 10 µm). (**c**) Internalization of rLj-RGD4 peptide into cells (Zeiss laser scanning confocal microscope; Oberkochen, Baden-Wurttemberg, Germany, 400×; scale: 10 µm). (**d**) Effect of rLj-RGD4 peptide on cells after treating with inhibitors filipin III, NaN_3,_ and cytochalasin D (Zeiss laser scanning confocal microscope 400×; scale: 10 µm).

We used the PBS-treated group as the blank control and rLj-RGD4 peptide-treated groups as experimental groups to investigate the inhibitory effects of the rLj-RGD4 peptide at varying concentration gradients on B16 cell proliferation. The cells were treated with PBS or rLj-RGD4 peptide for 24 h. The results demonstrated (Figure 7a) that the inhibitory effect on cell proliferation was enhanced with increasing rLj-RGD4 peptide concentration, indicating a dose-dependent suppression of B16 cell proliferation by the rLj-RGD4 peptide. The half-maximal inhibitory concentration (IC_50_) was 9.6 µM.

The PBS-treated group was used as the blank control; B16 cell proliferation inhibition was morphologically observed following treatment with 4.8, 9.6, and 19.2 µM rLj-RGD4 peptide in experimental groups. As shown in Figure 7b, control cells maintained a high density and normal spindle-shaped morphology after 24 h of culture. In contrast, treated B16 cells exhibited dose-dependent reductions in cell size and density, with progressive transformation from spindle-shaped to round shapes as the peptide concentration increased. These findings indicate that rLj-RGD4 disrupted B16 cell morphology and suppressed proliferation in a concentration-dependent manner.

#### 2.3.2. Mechanisms and Pathways of rLj-RGD4 Peptide Action on B16 Cells

We used the Alexa Fluor™ 488 Microscale Protein Labeling Kit to conjugate the rLj-RGD4 peptide with a green fluorescent dye, and its interaction with B16 cells was visualized in real-time via confocal microscopy. As shown in Figure 7c, immediately after peptide addition, fluorescent signals were detected primarily around B16 cells without obvious intracellular localization. Over time, green fluorescence gradually accumulated within the cytoplasm, coinciding with progressive cell shrinkage and enhanced nuclear contour clarity, indicating nuclear pyknosis, a hallmark of cellular apoptosis. These observations demonstrate that rLj-RGD4 undergoes active internalization into B16 cells, suggesting that its antitumor activity may be mediated through intracellular pathways following cellular uptake.

To investigate the internalization pathway of rLj-RGD4 peptide, B16 cells were treated with three distinct endocytic inhibitors—filipin III (lipid raft disruption), NaN_3_ (general endocytosis inhibition), and cytochalasin D (macropinocytosis and actin rearrangement inhibition)—before exposure to green fluorescently labeled rLj-RGD4 peptide (Alexa Fluor™ 488 Microscale Protein Labeling Kit). Nuclei were counterstained with Hoechst 33258 to visualize the cellular architecture. As shown in Figure 7d, treatment with filipin III or NaN_3_ for 1 h did not prevent peptide internalization, as fluorescent signals were still detected in the cytoplasm. Notably, cytochalasin D pretreatment completely abrogated intracellular peptide localization, with fluorescence retained exclusively at the cell periphery. These results indicate that rLj-RGD4 peptide internalization into B16 cells is predominantly mediated by macropinocytosis and actin rearrangement-dependent pathways, providing mechanistic insight into its apoptotic induction mechanism.

#### 2.3.3. Effect of rLj-RGD4 Peptide on Cell Surface EGFR in B16 Cells

**Figure 8:**
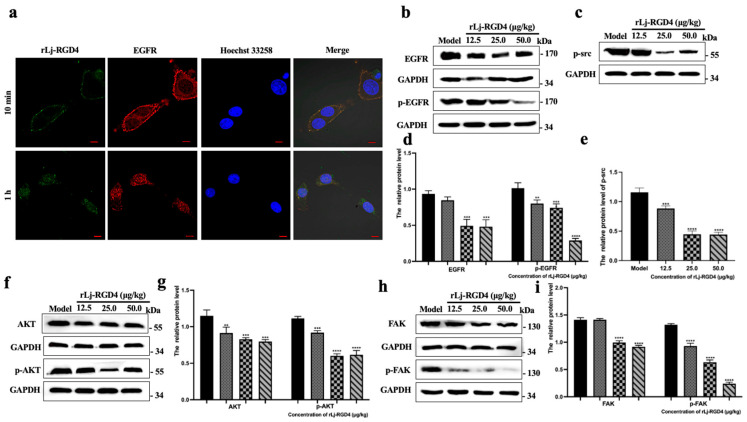
(**a**) The rLj-RGD4 peptide co-localized with EGFR (Zeiss laser scanning confocal microscope; Oberkochen, Baden-Wurttemberg, Germany, 400×; scale: 10 µm). (**b**) The protein level of EGFR and p-EGFR in tumor tissues. (**c**) Protein level of p-src in tumor tissues. (**d**) The relative gray value of EGFR and p-EGFR in tumor tissues of each group, GAPDH was used as a control; ** *p* < 0.01, *** *p* < 0.001, **** *p* < 0.0001. (**e**) The relative gray value of p-src in tumor tissues of each group, GAPDH was used as a control; *** *p* < 0.001, **** *p* < 0.0001. (**f**) The protein levels of AKT and p-AKT in tumor tissues. (**g**) The relative gray value of AKT and p-AKT in tumor tissues of each group, GAPDH was used as a control. ** *p* < 0.01, *** *p* < 0.001, **** *p* < 0.0001. (**h**) The protein levels of FAK and p-FAK in tumor tissues. (**i**) The relative gray value of FAK and p-FAK in tumor tissues of each group, GAPDH was used as a control. **** *p* < 0.0001.

We next investigated the interaction between the rLj-RGD4 peptide and cell surface EGFR, for which B16 cells were treated with 9.6 µM rLj-RGD4 peptide labeled with Alexa Fluor™ 488 (green fluorescence), whereas EGFR was stained with a red fluorescent antibody. Nuclei were counterstained with Hoechst 33258 to visualize nuclear regions. As shown in Figure 8a, 10 min post-treatment, the rLj-RGD4 peptide exhibited significant co-localization with EGFR at the cell membrane, indicating a direct interaction between the two molecules. The rLj-RGD4 peptide was detected in the cytoplasm and nucleus, accompanied by internalization of EGFR into the cytoplasm when the incubation time was extended to 1 h. These results demonstrate that the rLj-RGD4 peptide binds to cell surface EGFR and transiently induces receptor internalization, while prolonged treatment leads to inhibition of EGFR internalization, as shown by Western blotting. This dynamic interaction may modulate intracellular signaling.

EGFR (ErbB1/HER1) regulates cell proliferation, survival, migration, invasion, and apoptosis through signaling pathways involving serine/threonine kinases, such as AKT, FAK, and Src. Overexpression or mutation of *EGFR* and its ligands in most malignant tumors has established EGFR as a critical target for cancer therapy [18]. Building on previous evidence that rLj-RGD4 interacts with EGFR and induces its internalization, Western blotting was performed to examine EGFR and downstream signaling proteins in the tumor tissues of B16 melanoma-bearing mice treated with rLj-RGD4.

As shown in Figure 8b,d, compared with the model group, low, medium, and high doses of rLj-RGD4 peptide significantly downregulated EGFR expression in a dose-dependent manner, with similar trends observed for p-EGFR. Figure 8h,i demonstrate that FAK and p-FAK levels were significantly reduced in a dose-dependent manner across all rLj-RGD4 treatment groups relative to the model group. In contrast, Figure 8f,g show that AKT expression was slightly decreased at the low rLj-RGD4 dose; however, it was significantly upregulated at medium and high doses in a dose-dependent manner, whereas p-AKT levels were consistently downregulated. Figure 8c,e further indicate that p-Src expression was markedly suppressed in all the treatment groups in a dose-dependent manner.

Collectively, Western blotting results suggested that the rLj-RGD4 peptide inhibited EGFR internalization, thereby blocking downstream signaling through the FAK, AKT, and Src pathways. The suppression of phosphorylated proteins and modulation of key signaling nodes possibly induce apoptosis while inhibiting tumor cell proliferation, migration, and survival, ultimately mediating the antitumor effects of the rLj-RGD4 peptide.

## 3. Discussion

rLj-RGD4 is a genetically truncated RGD peptide derived from the parental protein rLj-RGD3, with a molecular weight reduced from 13.5 kDa to 6.27 kDa and four tandem RGD motifs. Previous studies have confirmed that rLj-RGD3 exerts inhibitory effects on tumor cell proliferation, migration, and invasion by targeting integrins, but its relatively large molecular weight raises potential immunogenicity risks at high doses. As a truncated derivative, rLj-RGD4 retains the core RGD functional structure while significantly reducing molecular size, which is consistent with the design strategy of most peptide drugs to improve safety and reduce immunogenicity. Our prior in vitro experiments demonstrated that rLj-RGD4 effectively inhibits proliferation, migration, and invasion of B16 melanoma cells and disrupts actin cytoskeleton organization, but its in vivo antitumor efficacy and detailed molecular mechanisms remained unclear. Therefore, this study used a B16 melanoma xenograft mouse model to systematically evaluate the in vivo activity and mechanism of rLj-RGD4, providing a preclinical basis for its further development.

In the present study, rLj-RGD4 was administered intraperitoneally at 12.5, 25.0, and 50.0 μg/kg for 14 consecutive days. Results showed that rLj-RGD4 dose-dependently suppressed tumor volume and weight, with inhibition rates of 21.60–89.26% for volume and 41.03–86.51% for weight. Notably, the high-dose group (50.0 μg/kg) exhibited stronger antitumor efficacy than the positive control cyclophosphamide (20.0 mg/kg), while causing no significant body weight loss or obvious toxic signs, in contrast to the obvious systemic toxicity of CTX. This finding is consistent with previous reports that small-molecule RGD peptides show high antitumor potency at microgram-level doses with better safety than traditional chemotherapeutics. Compared with the parental molecule rLj-RGD3, which requires 160 μg/kg to achieve effective tumor inhibition, rLj-RGD4 achieves comparable or stronger effects at a lower dose, supporting that truncation optimization improves potency while reducing the risk of immunogenicity, which aligns with the development trend of RGD-based peptide drugs toward smaller size and higher specificity.

Histopathological examinations (HE, Masson’s trichrome, Hoechst, and TUNEL) consistently confirmed that rLj-RGD4 induces severe tumor cell apoptosis and tissue fibrosis. These morphological changes are highly consistent with the typical characteristics of RGD peptide-induced tumor inhibition reported in previous studies. Western blot analysis further revealed that rLj-RGD4 simultaneously activates multiple apoptotic pathways: (1) the mitochondrial pathway, marked by upregulated Bax, downregulated Bcl-2, increased Bax/Bcl-2 ratio, and activated caspase-9 and caspase-3; (2) the death receptor pathway, characterized by cleaved caspase-8; and (3) the MAPK pathway, showing inhibited p-Erk1/2 and activated p-p38. This multi-pathway pro-apoptotic pattern is not commonly seen in single-target RGD peptides. Most reported RGD peptides mainly act on integrins to regulate single apoptotic signaling, while rLj-RGD4 triggers a synergistic network of mitochondrial, death receptor, and stress-activated MAPK pathways, which explains its strong in vivo antitumor effect. This multi-pathway regulatory feature is similar to the mechanism of some multi-target antitumor peptides, suggesting that rLj-RGD4 has potential advantages in overcoming drug resistance caused by single-pathway blockade.

A key finding of this study is that rLj-RGD4 targets EGFR and its downstream signaling, forming a dual-target mechanism against integrins and EGFR [21,22,23]. Confocal microscopy showed that rLj-RGD4 colocalizes with EGFR on the cell membrane and promotes EGFR internalization. Western blot demonstrated dose-dependent downregulation of EGFR, p-EGFR, and downstream p-FAK, p-AKT, and p-Src. These results indicate that rLj-RGD4 directly binds EGFR, blocks its phosphorylation, and inhibits the pro-survival signaling axis. This dual-target mechanism is consistent with recent studies showing crosstalk between integrins and EGFR in melanoma progression. Many RGD peptides have been reported to indirectly affect EGFR signaling by inhibiting integrins, but few can directly bind and internalize EGFR like rLj-RGD4. This dual-target property makes rLj-RGD4 superior to single-integrin-targeting RGD peptides and provides a new strategy for developing multi-target anti-melanoma peptides.

Endocytosis mechanism experiments showed that rLj-RGD4 enters B16 cells mainly through actin-dependent macropinocytosis, as its internalization was significantly blocked by cytochalasin D but not by filipin III or NaN_3_. This uptake pathway is consistent with the internalization characteristics of some small-molecule antitumor peptides and helps rLj-RGD4 exert intracellular pro-apoptotic effects. In contrast, many RGD peptides only act on cell-surface integrins without effective internalization, limiting their in vivo efficacy. The macropinocytosis pathway of rLj-RGD4 supports its ability to penetrate tumor cells and regulate intracellular signaling, which is an important molecular basis for its strong in vivo activity.

Regarding safety, the low molecular weight (6.27 kDa) of rLj-RGD4 reduces the number of antigenic epitopes. The parental rLj-RGD3 showed no immunogenicity at therapeutic doses and only mild immunogenicity at more than 8-fold doses. Since the highest effective dose of rLj-RGD4 (50.0 μg/kg) is far below the immunogenic threshold of rLj-RGD3, rLj-RGD4 is expected to have low immunogenic risk. This inference is supported by studies showing that peptide drugs below 10 kDa generally have low immunogenicity. However, formal immunogenicity evaluation still needs to be carried out in future studies.

## 4. Materials and Methods

### 4.1. Expression and Purification of Recombinant Peptide rLj-RGD4

Gene synthesis and cloning of rLj-RGD4, the recombinant mutant truncated peptide, were conducted by TaKaRa Bio Inc. (Dalian, China). The recombinant plasmid pET23b-RGD4 and recombinant expression strain BL21 harboring rLj-RGD4 were preserved in our laboratory under appropriate storage conditions to maintain their integrity and functionality for subsequent experiments.

### 4.2. Antitumor Effect of rLj-RGD4 Peptide in B16 Melanoma-Bearing Mice

#### 4.2.1. Cell Culture

The B16 mouse melanoma cell line was kindly provided by Prof. Pixu Liu of Dalian Medical University. Frozen B16 cells were thawed and inoculated into cell culture flasks, subsequently cultured at 37 °C in a humidified incubator with 5% CO_2_. When the cells reached approximately 80% confluence, they were passaged at a ratio of 1:3 using trypsin–EDTA digestion, and subcultured every 3 days until sufficient cell numbers were obtained for experiments.

#### 4.2.2. Establishment of B16 Melanoma Tumor-Bearing Mouse Model

Forty SPF-grade male BALB/c mice (3–4 weeks old, body weight 20 ± 2 g) were purchased from the Laboratory Animal Center of Dalian Medical University (license number: SCXK(Liao)2016-0008) and housed under SPF conditions with sterilized feed at a controlled room temperature of 21 ± 1 °C. B16 cells were digested with trypsin, centrifuged to remove the culture medium, and resuspended in fresh serum-free medium to prepare a cell suspension at a concentration of 5 × 10 cells/mL. A 0.2 mL aliquot of the cell suspension was subcutaneously inoculated into the right axillary region of each mouse to establish a tumor-bearing model.

#### 4.2.3. Effect of rLj-RGD4 Peptide on Tumor Growth in B16 Melanoma Tumor-Bearing Mice

Tumor length (a) and width (b) were measured daily after successful establishment of the B16 tumor-bearing mouse model. When the maximum tumor diameter reached 4–6 mm, 40 mice were randomly assigned into five groups (*n* = 8 per group): a model group receiving intraperitoneal (i.p.) injections of saline twice daily, a positive control group administered i.p. cyclophosphamide (CTX, 20.0 mg/kg) once daily, and three rLj-RGD4 peptide groups treated with i.p. doses of 12.5 μg/kg, 25.0 μg/kg, and 50.0 μg/kg twice daily, with a standardized administration volume of 0.2 mL/g body weight for all groups over 14 consecutive days. Daily monitoring included recording body weight and tumor dimensions to calculate the tumor volume using Equation (1). The mice were euthanized, tumor tissues were excised to measure weight and volume, and tumor inhibition rates were calculated using Equations (2) and (3) based on comparisons with the model group at the end of the treatment period.

(1) $$Tumor\;volume=0.5\times a\times b^{2}$$

(2) $$Tumor\;volume\;inhibition\;rate=\frac{{Tumor\;volume}_{control}-{Tumor\;volume}_{experimental\;}}{{Tumor\;volume}_{control}}\times 100\%$$

(3) $$Tumor\;weight\;inhibition\;rate=\frac{{tumor\;weight}_{control}-{tumor\;weight}_{experimental\;}}{{tumor\;weight}_{control}}\times 100\%$$

#### 4.2.4. Paraffin Sectioning

Tumor tissues were fixed for 24 h and dehydrated using a graded ethanol series (80% and 90% for 40 min; 95% and 100% twice for 20 min each). Subsequent clearing was performed twice in xylene/ethanol (1:1) for 15 min each, followed by pure xylene (15 min each). Tissues were infiltrated with xylene/paraffin (Solarbio; Beijing, China) for 15 min and pure paraffin twice (30 min each), embedded in molten paraffin, and rapidly cooled. After trimming, 4 μm sections were cut using a microtome and labeled for further analysis.

#### 4.2.5. Hematoxylin and Eosin (H&E) Staining

The tissue sections were deparaffinized and stained with hematoxylin (Solarbio) for 20 min, followed by washing in distilled water until a blue color appeared. Differentiation was performed in 1% acidic alcohol for several seconds, followed by rinsing with distilled water. The sections were dehydrated through a graded ethanol series (70%, 80%, and 95%, 5 min each) and counterstained using a 0.5% eosin ethanol solution. Finally, the sections were dehydrated, cleared, mounted with neutral resin, and examined under an inverted microscope.

#### 4.2.6. Masson Trichrome Staining

Deparaffinized sections were stained with Weigert’s iron hematoxylin (Solarbio) (1:1 mixture of solutions A/B, 5–10 min), differentiated in acidic alcohol (5–15 s), and stained with Masson’s solution (3–5 min). The samples were rinsed in distilled water and stained with ponceau–fuchsin (5–10 min), treated with phosphomolybdic acid (1–2 min), and counterstained with aniline blue (1–2 min) and a weak acid working solution (1:2 dilution) washes between steps. Finally, the sections were dehydrated, cleared, mounted with neutral resin, and examined under an inverted microscope.

#### 4.2.7. Hoechst 33258 Staining

Deparaffinized sections were washed twice with PBS (3 min each) and stained with 0.5 mL of Hoechst 33258 solution (Beyotime, Shanghai, China) for 5 min. The staining solution was removed, and the samples were rinsed twice with PBS (3 min each), mounted with an antifade medium under coverslips sealed with nail polish, and examined using a fluorescence microscope.

#### 4.2.8. TUNEL Assay for Apoptosis Detection

Deparaffinized sections were treated with DNase-free proteinase K (20 μg/mL; 37 °C, 25 min), followed by three PBS washes. The TUNEL reaction mixture (Beyotime) was prepared according to the manufacturer’s protocol and applied to sections for 90 min at 37 °C in the dark. After three additional PBS washes, the samples were mounted with an antifade medium under coverslips sealed with nail polish and examined using fluorescence microscopy.

#### 4.2.9. Western Blotting

Tumor tissues were minced with sterile scissors and homogenized in radioimmunoprecipitation lysis buffer containing PMSF (1000:1 ratio, 100–200 μL per 20 mg tissue). After 30 min of incubation for complete lysis, the homogenate was centrifuged at 12,000 rpm for 5 min at 4 °C, and the supernatant was collected as a protein extract. The protein concentration was determined using the Bradford assay (Coomassie Brilliant Blue G-250, Beyotime). The protein samples were mixed with 5× loading buffer (4:1 ratio), boiled for 5 min, aliquoted, and stored at −80 °C.

Protein samples and markers were loaded onto SDS–PAGE gels and electrophoresed at 80–120 V until bromophenol blue reached the bottom. The target protein bands were excised and transferred to polyvinylidene fluoride (PVDF) membranes (pre-activated with methanol) using a semi-dry transfer system (1 kDa/min) with filter paper sandwiches. After blocking for 1 h at room temperature, membranes were incubated with primary antibodies (1:1000 dilution in PBST/blocking buffer) overnight at 4 °C, followed by incubation with secondary antibodies (1:5000) for 2 h at room temperature. Following extensive PBST washes (4 × 10 min each), protein signals were detected using the ECL substrate and quantified using AlphaView SA software (Specialized Analysis, 3.4.0.729). Statistical analysis was performed using Student’s *t*-test (mean ± SEM, *n* = 3), with *p* < 0.05 considered significant.

The following antibodies were used: GAPDH (Cell Signaling Technology, Beverly, MA, USA, 1:1000), Bax (Cell Signaling Technology, 1:1000), Bcl-2 (Cell Signaling Technology, 1:1000), caspase-8 (Cell Signaling Technology, 1:1000), cleaved caspase-8 (Cell Signaling Technology, 1:1000), caspase-9 (Cell Signaling Technology, 1:1000), cleaved caspase-9 (Cell Signaling Technology, 1:1000), caspase-3 (Cell Signaling Technology, 1:1000), cleaved caspase-3 (Cell Signaling Technology, 1:1000), PARP (Cell Signaling Technology, 1:1000), phospho-p44/42 MAPK (Cell Signaling Technology, 1:2000), phospho-p38 MAPK (Cell Signaling Technology, 1:1000), EGFR (Cell Signaling Technology, 1:1000), phospho-EGFR (Cell Signaling Technology, 1:1000), FAK (Cell Signaling Technology, 1:1000), phospho-FAK (Cell Signaling Technology, 1:1000), Akt (Cell Signaling Technology, 1:1000), phospho-Akt (Cell Signaling Technology, 1:1000), phospho-Src (Cell Signaling Technology, 1:1000), horseradish peroxidase-conjugated goat anti-rabbit (Zsbio, Beijing, China, 1:5000), and horseradish peroxidase-conjugated goat anti-mouse (Zsbio, 1:5000).

### 4.3. Mechanisms and Pathways of rLj-RGD4 Peptide Action on B16 Cells and Its Effect on EGFR

#### 4.3.1. Cell Proliferation and Cytotoxicity Assay

Cell viability was assessed using a CCK-8 kit (Beyotime; Shanghai, China), in which WST-8, a water-soluble MTT analog, was reduced by mitochondrial dehydrogenases in viable cells to generate orange-yellow formazan. The optical density (OD) correlates with the number of metabolically active cells. B16 cells were plated in 96-well plates, incubated with 3 ng/mL bFGF for 24 h (37 °C), and treated with serially diluted rLj-RGD4 peptide or PBS (control) for 24 h. CCK-8 reagent (10% *v*/*v*) was added to each well and incubated for 4 h. The absorbance was measured at 450 nm using a microplate reader. The proliferation inhibition rate was calculated as follows:

Inhibition rate (%) = [(OD_control_ − OD_experimental_)/OD_control_] × 100,

with triplicate independent experiments and data normalized to PBS-treated controls.

#### 4.3.2. Morphological Analysis by Giemsa Staining

Giemsa staining was performed using a dye mixture containing azure B and eosin Y (Beyotime). Acidic cytoplasmic proteins preferentially bind to eosin (red staining), whereas basic nuclear DNA interacts with azure B (blue-purple staining), enabling differential visualization of cellular compartments. Briefly, sterilized glass coverslips were placed in 6-well plates and seeded with B16 cells. When cells reached 70–80% confluency, they were treated with serially diluted rLj-RGD4 peptide or PBS for 24 h at 37 °C. The culture medium was aspirated, and the cells were fixed with Solution I (fixative) for 5 min, followed by staining with Solution II (Giemsa working solution) for 5–8 min. After a 30 s distilled water rinse, the coverslips were mounted on glass slides using a neutral resin and imaged under an inverted microscope.

#### 4.3.3. Fluorescently Labeled Proteins

Proteins were fluorescently labeled to perform confocal microscopy: 1 M sodium bicarbonate solution was prepared, and 45 μL of 1.15 mg/mL protein solution was mixed with 4.5 μL of 1 M sodium bicarbonate in a reaction tube (C). After adding 10 μL of deionized water to the Alexa Flour™488 TFP tube (Invitrogen™, Wathlam, MA, USA), 5 μL of the resulting dye solution was transferred to tube (C) and incubated at room temperature for 30 min. The gel resin in container (E) was gently resuspended, 800 μL was loaded into a spin filter, and centrifuged at 12,000 rpm for 15 s. Afterward, 50 μL of the reaction mixture was added to the resin center, and the filter (with the resin-rich side outward) was centrifuged at 12,000 rpm for 1 min to collect the fluorescently labeled protein.

B16 cells were seeded in confocal dishes. At ~70% confluency, the medium was replaced with 500 μL of fresh medium containing 9.6 μM fluorescently labeled rLj-RGD4 peptide, followed by 1 h incubation. All subsequent steps were protected from light. The cells were fixed with 4% paraformaldehyde at room temperature for 30 min, washed thrice with PBS (5 min per wash), blocked with 200 μL of diluted goat serum at 37 °C for 40 min, and the blocking solution was removed. Anti-EGFR antibody (1:50 dilution in blocking solution) was added and incubated at 37 °C for 1 h, followed by three washes with PBST (15 min per wash). A fluorescent secondary antibody (1:100 dilution in PBST) was added and incubated at 37 °C for 30 min. After four PBS washes (10 min per wash), the nuclei were stained with Hoechst 33258 for 5 min at room temperature, followed by three additional PBS washes (5 min per wash). Finally, a fluorescent anti-quenching mounting medium was applied, and images were captured under a laser confocal microscope at 600× magnification.

#### 4.3.4. Mechanistic Analysis of rLj-RGD4 Cellular Internalization Pathways

We next wanted to delineate the endocytic mechanisms of rLj-RGD4, for which B16 cells grown on sterilized glass coverslips (70–80% confluency in 6-well plates) were pretreated for 1 h at 37 °C with pathway-specific inhibitors: 5 μg/mL filipin III (lipid raft-mediated endocytosis blockade), 10 mM NaN_3_ (energy-dependent endocytosis inhibition), or 10 μM cytochalasin D (macropinocytosis/actin remodeling suppression). Cells were subsequently exposed to 9.6 μM Alexa Fluor™ 488-conjugated rLj-RGD4 for 1 h, followed by fixation (4% paraformaldehyde, 10 min) and nuclear counterstaining with Hoechst 33258 (5 μg/mL, 5 min). After washing with PBS (3 × 3 min), the coverslips were mounted using antifade medium and imaged under a fluorescence microscope (Carl Zeiss, Oberkochen, Germany).

## 5. Conclusions

This study demonstrates that the rLj-RGD4 peptide potently inhibits B16 melanoma growth in vivo by inducing tumor cell apoptosis, achieving significant antitumor efficacy at microgram-level doses (12.5–50.0 μg/kg)—notably more potent than the milligram-level dosage of cyclophosphamide (20.0 mg/kg). Its low molecular weight (6.27 kDa) minimizes the immunogenicity risk, supported by the dosage–safety profile of its parental protein rLj-RGD3, and aligns with the structural features of peptides with reduced antigenic epitopes.

The rLj-RGD4 peptide induced apoptosis via multi-targeted regulation of survival pathways, including death receptor, mitochondrial-mediated, and MAPK pathways. Confocal microscopy and inhibitor studies revealed its internalization into B16 cells via macropinocytosis and actin rearrangement. Immunofluorescence and Western blot analyses further demonstrated that rLj-RGD4 interacts with cell surface EGFR, induces its internalization, and suppresses EGFR signal transduction, establishing a dual-target mechanism involving integrins and EGFR.

Collectively, these findings highlight rLj-RGD4 as a promising antitumor candidate with high efficiency, low toxicity, and a novel multimechanistic action profile. Future translational research should prioritize preclinical safety evaluations, particularly immunogenicity assessments, to facilitate their development as clinical anticancer agents.

## Figures and Tables

**Table 1:** Effect of 4 peptides on tumor volume in B16 melanoma-bearing mice.

Group	Tumor Volume (mm^3^)	Inhibition Rate (%)
Model	2305.65 ± 1029.62	--
CTX (20.0 mg/kg)	803.61 ± 229.47 ***	64.32
rLj-RGD4 (12.5 μg/kg)	1975.02 ± 642.86 ***	21.60
rLj-RGD4 (25.0 μg/kg)	566.18 ± 410.55 ***	77.19
rLj-RGD4 (50.0 μg/kg)	356.46 ± 250.77 ***	89.26

($\bar{X}$ ± SD, *n* = 6, compared with control, *** *p* < 0.001).

**Table 2:** Effect of rLj-RGD4 peptide on tumor weight in B16 melanoma-bearing mice.

Group	Tumor Weight (g)	Inhibition Rate (%)
Model	2.47 ± 0.63	--
CTX (20.0 mg/kg)	0.50 ± 0.18 *	80.00
rLj-RGD4 (12.5 μg/kg)	1.46 ± 0.40 *	41.03
rLj-RGD4 (25.0 μg/kg)	0.42 ± 0.24 **	81.90
rLj-RGD4 (50.0 μg/kg)	0.35 ± 0.23 **	86.51

($\bar{X}$ ± SD, *n* = 6, compare with control, * *p* < 0.05, ** *p* < 0.01).

## Data Availability

The original contributions presented in this study are included in the article. Further inquiries can be directed to the corresponding author.
